# Tumor Initiating Cells in Esophageal Squamous Cell Carcinomas Express High Levels of CD44

**DOI:** 10.1371/journal.pone.0021419

**Published:** 2011-06-24

**Authors:** Jiang-Sha Zhao, Wen-Jie Li, Di Ge, Pei-Jing Zhang, Jing-Jing Li, Chun-Lai Lu, Xiao-Dan Ji, Dong-Xian Guan, Hong Gao, Li-Yan Xu, Eng-Ming Li, Harmik Soukiasian, H. Phillip Koeffler, Xiao-Fan Wang, Dong Xie

**Affiliations:** 1 Laboratory of Molecular Oncology, Institute for Nutritional Sciences, Shanghai Institutes for Biological Sciences, Chinese Academy of Sciences and Graduate School of Chinese Academy of Sciences, Shanghai, China; 2 College of Public Health, Zhengzhou University, Zhengzhou, China; 3 Department of Thoracic Surgery, Zhongshan Hospital, Shanghai Medical College, Fudan University, Shanghai, China; 4 Department of Biochemistry and Molecular Biology, Medical College of Shantou University, Shantou, China; 5 Department of Surgery, Cedars-Sinai Medical Center, University of California Los Angeles (UCLA) School of Medicine, Los Angeles, California, United States of America; 6 Cancer Science Institute of Singapore, National University of Singapore, Singapore; 7 Department of Pharmacology and Cancer Biology, Duke University Medical Center, Durham, North Carolina, United States of America; The University of Texas M.D Anderson Cancer Center, United States of America

## Abstract

**Background:**

Esophageal Squamous Cell Carcinoma (ESCC) is a major subtype of esophageal cancer causing significant morbility and mortality in Asia. Mechanism of initiation and progression of this disease is unclear. Tumor initiating cells (TICs) are the subpopulation of cells which have the ability to self-renew, as well as, to drive initiation and progression of cancer. Increasing evidence has shown that TICs exist in a variety of tumors. However, the identification and characterization of TICs in esophageal carcinoma has remained elusive.

**Methodology/Principal Findings:**

to identify TICs in ESCC, ESCC cell lines including two primary cells were used for screening suitable surface marker. Then colony formation assay, drug resistant assay and tumorigenicity assay in immune deficient mice were used to characterize TICs in ESCC. We found that just the CD44 expression correlated with tumorigenicity in ESCC cell lines. And then induced differentiation of ESCC cells by all-trans retinoic acid treatment led to decreased expression of CD44. The FACS isolated cell subpopulations with high CD44 expression showed increased colony formation and drug resistance *in vitro*, as well as significantly enhanced tumorigenicity in NOD/SICD mice, as compared to the low expressing CD44 ESCC cells.

**Conclusions/Significance:**

our study has discovered a novel TIC surface marker, CD44, which can be utilized to enrich efficiently the TICs in ESCC. These findings will be useful for further studies of these cells and exploring therapeutic approaches.

## Introduction

Esophageal cancer is one of the most common malignancies throughout the world, ranking eighth in incidence [1]. It can be pathologically classified into two major subtypes, Esophageal Adenocarcinomas (EAC) and Esophageal Squamous Cell Carcinomas (ESCC). ESCC especially occurs in developing countries, and is particularly prevalent in China and other counties in Asia [1],[2]. Although progress has occurred in the diagnosis and treatment of ESCC, the rate of mortality from this disease has not improved significantly [3], [4]. Lack of advances probably reflects both not making the diagnosis of the disease until an advanced stage and a poor understanding of the cellular and molecular mechanism underlying initiation and progression of ESCC.

Tumor initiating cells (TICs) or cancer stem cells (CSCs) are defined as a subset of cells in tumors with the capability to self-renew and differentiate, thus they can either initiate or maintain a tumor. TICs also exhibit greater resistance to conventional chemo- and radio-therapies than more differentiated tumor cells [5]. Therefore, TICs are crucial targets for cancer therapy.

After its first detection in leukemia, TICs have been widely discovered in solid tumors, including breast, colon, glioma, prostate, liver and melanoma [6], [7], [8], [9], [10], [11], [12]; however, these cells have remained elusive in ESCC. In the present study, we have identified CD44 as a TIC surface marker in ESCC, and successfully enriched TICs based upon their elevated expression of CD44. Discovery of this novel TIC marker in ESCC will not only benefit the investigation of ESCC, but will also provide the appropriate target cell for development of effective therapeutic strategies for ESCC.

## Results

### Expression of CD44 Correlates with the Tumorigenicity of ESCC Cell Lines

**Figure 1:**
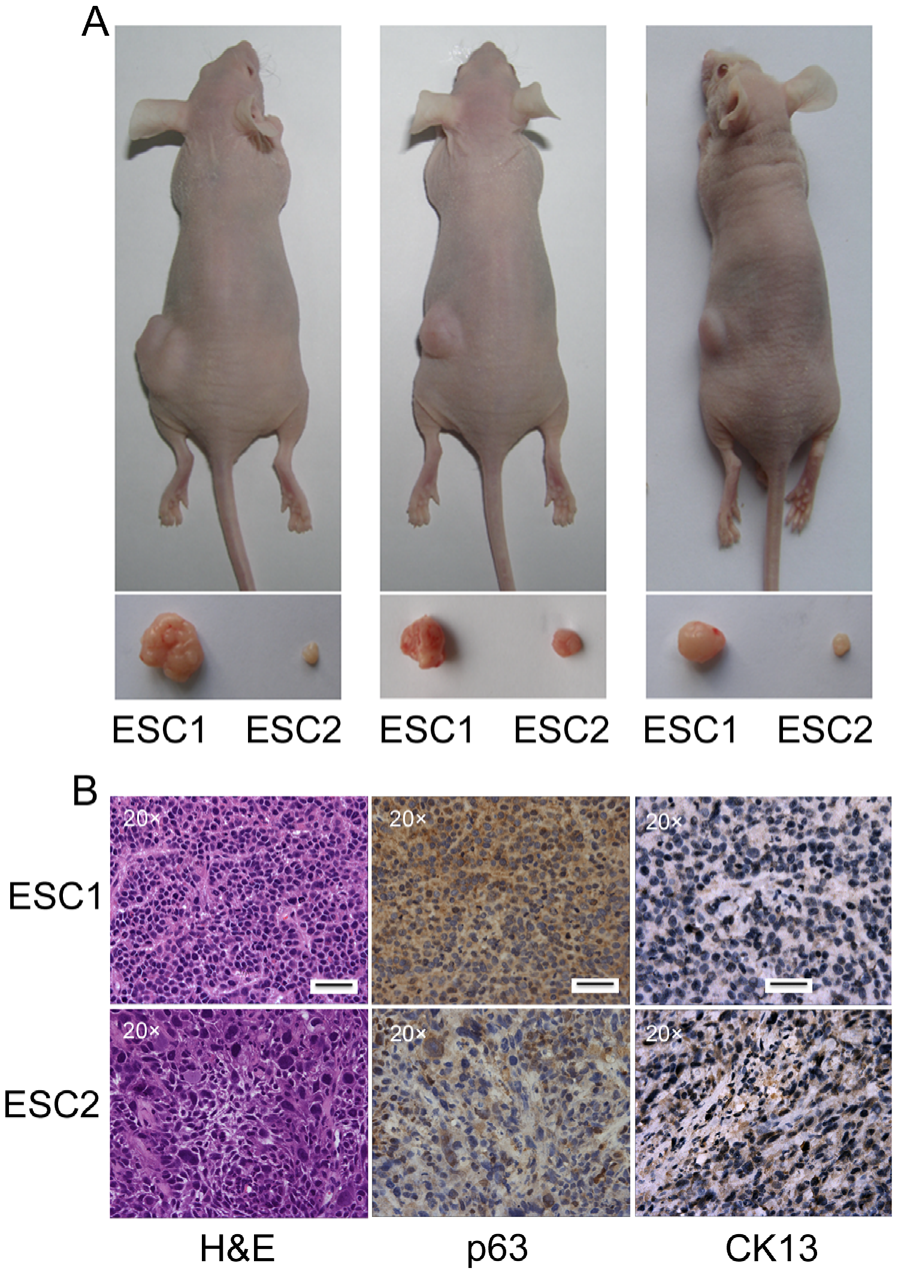
ESC1 is more tumorigenic than ESC2 cells. (A) 5×10^5^ of ESC1 or ESC2 cells were s.c. injected into nude mice. 3 weeks after cell injection, all mice were sacrificed; tumors were isolated and their weights were compared. (B) Tumors derived from both ESC1 and ESC2 were paraffin embedded. H&E staining was performed on these samples. Representative IHC analyses of p63 (a stemness marker), and CK13 (a differentiation marker) in ESC1 and ESC2 tumors. Scale bar: 50 micrometer.

**Figure 2:**
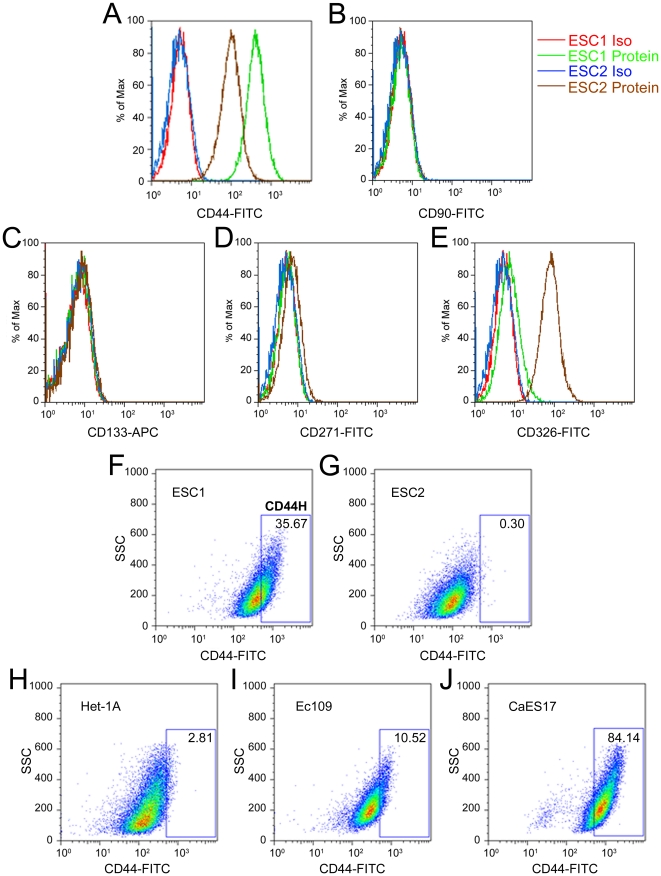
Expression of cell surface protein on ESCC cells. (A–E) measurement of the expression of CD44 (A), CD90 (B), CD133 (C), CD271 (D) and CD326 (E) in ESC1 and ESC2 cells by flow cytometry analysis. (F–J) Representative flow cytometry results examining the expression of CD44 in ESC1 (F), ESC2 (G), Het-1A (H), Ec109 (I) and CaES17 (J) cells. Using the comparative level of expression of CD44 in ESC1 versus Het-1A, we defined CD44 highly expressed subpopulation cells within the ESC1 line as CD44H (High) cells, and the remainder of the cell population as CD44L (Low) (F). ESC1 Iso (red line): ESC1 cells stained with isotype control antibody; ESC1 Protein (green line): ESC1 cells stained with candidate surface protein antibody; ESC2 Iso (blue line): ESC2 cells stained with isotype control antibody; ESC2 Protein (brown line): ESC2 cells stained with candidate surface protein antibody; FITC: fluorescein isothiocyanate; APC: allophycocyanin; SSC: side scatter.

To identify TICs marker for ESCC, we initially analyzed the expression pattern of several candidate molecules in the two primary ESCC cells (ESC1 and ESC2), which were isolated from ESCC specimens and demonstrated significant difference in tumorigenicity (Figure 1A). Based on the previous studies of TICs in other solid carcinomas, the following cell surface proteins were selected: CD44, CD90, CD133, CD271 and CD326. When ESC1 and ESC2 were analyzed by flow cytometry, only CD44 expression was correlated with tumorigenicity. Although both cell lines expressed the protein, the expression level of CD44 was much higher in ESC1, which is the more tumorigenic ESCC cell (Figure 2A, F and G). Other candidates were either undetectable in both cells (CD90, CD133 and CD271) or inversely correlated with tumorigenicity (CD326) (Figure 2B∼E).

The differential expression of CD44 on ESC1 and ESC2 cells prompted us to analyze its expression in Het-1A, an immortalized esophageal epithelial cell line as well as other ESCC cell lines to examine further the correlation between expression of CD44 and tumorigenicity. The expression level of CD44 in Het-1A was similar to the low expressing ESC2 cells (Figure 2H). Two other ESCC cell lines, Ec109 and CaES17 were CD44-positive, and displayed higher expression levels compared to Het-1A and ESC2 cells (Figure 2I, J). Using the comparative level of expression of CD44 in ESC1 versus Het-1A, we defined CD44 highly expressed subpopulation cells within the ESC1 line as CD44H (High) cells and the remainder of the cell population as CD44L (Low) (Figure 2F).

### Expression of CD44 in ESCC Specimens

**Figure 3:**
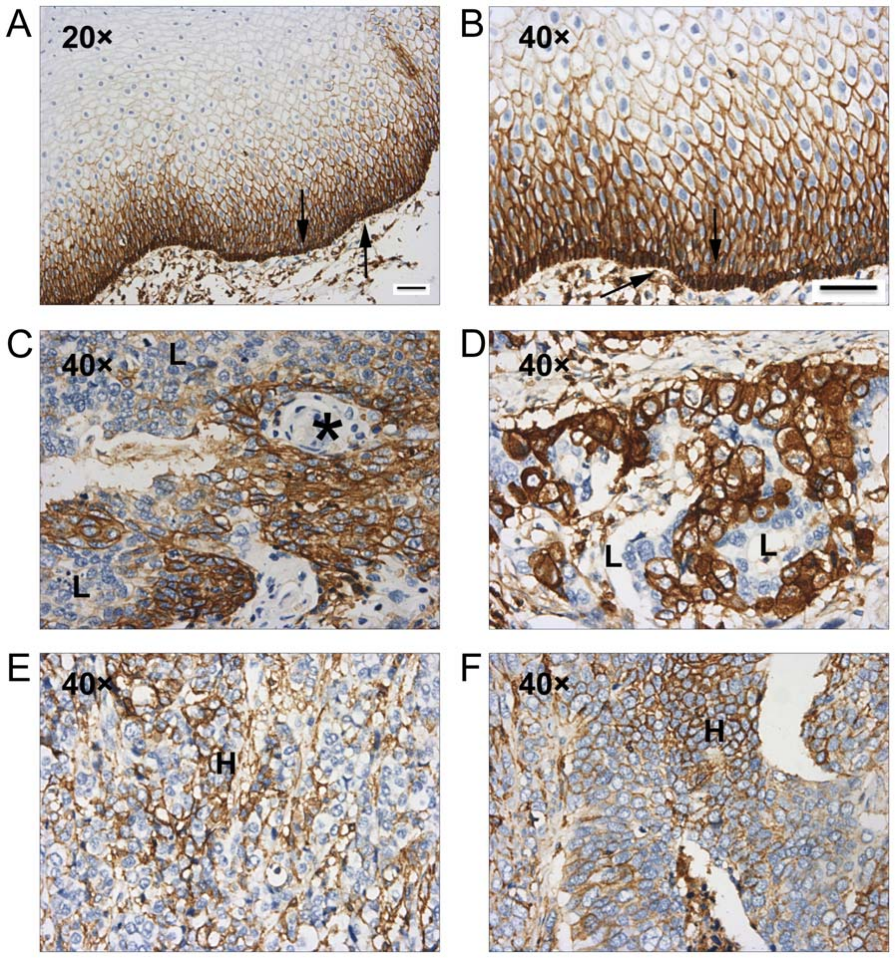
Expression of CD44 in ESCC specimens. (A) Representative IHC analyses of CD44 in normal esophageal tissues. (B) Magnification of (A) demonstrated that the expression of CD44 was in the basal layer (indicated by arrow). (C–F) Representative IHC analyses of CD44 in ESCC tissues. (C–D) showed specimens with strong expression of CD44 and (E–F) showed cases with weak expression of CD44. * indicated keratin pearl, a structure which represents terminal differentiation of the cancer cells; Letter “**L**”, cells exhibiting lower expression level of CD44 in (C–D); letter “**H**”, cells exhibiting higher expression of CD44 in (E–F). Scale bar: 50 micrometer.

To investigate whether the potential ESCC TICs with high expression level of CD44 can be detected in clinical specimens, we examined CD44 expression in 171 paraffin-embedded ESCC tissues by IHC staining. In normal esophageal tissues, CD44 was found to be heterogeneously expressed. Among all the ESCC specimens, 30% (52/171) were either negative or weakly positive for CD44, and 70% (119/171) were CD44 strongly positive. The strongest expression of CD44 was found in the basal layer where normal esophageal stem cells are localized (Figure 3A, B). Although no association was found between CD44 expression and clinicopathological parameters of ESCC patients, except the degree of cellular differentiation (Table 1), heterogeneous expression of CD44 was noted in most specimens. Keratin pearls are structures that occur during terminal differentiation of ESCC cells; these cells were either weakly positive or negative for CD44 expression (Figure 3C). For most samples, most of the cells were uniformly strong expressers of CD44; however, a minority of cells within these samples had low expression of CD44 (Figure 3C, D). A similar heterogeneity of expression occurred in specimens with low average weak expression of CD44; nevertheless, some of cells strongly expressed CD44 (Figure 3E, F). These results suggest that potential ESCC TICs with high expression level of CD44 may exist in clinical ESCC samples.

**Table 1:** Relationship between clinicopathological features and CD44 expression.

Parameters	CD44 expression level		p-value*
	Negative or low	High	
Age			0.733
<55	18 (29%)	45 (71%)	
≥55	34 (31%)	74 (69%)	
Gender			0.338
Male	36 (28%)	92 (72%)	
Female	16 (37%)	27 (63%)	
Regional lymph nodes			1.000
N0	28 (31%)	63 (69%)	
N1	24 (30%)	56 (70%)	
TNM staging			1.000
I/IIa	28 (31%)	63 (69%)	
IIb/III/IV	24 (30%)	56 (70%)	
Grade of differentiation			0.001
Well	6 (13%)	42 (87%)	
Moderate	35 (34%)	68 (66%)	
Poor	11 (55%)	9 (45%)	

The IHC procedure was done as described previously [40]. The intensity of positive staining for CD44 was evaluated as negative, weak staining, moderate staining and strong staining. For chi-square test, negative, weak and moderate staining for CD44 were considered as CD44-negative or -low expression, and strong staining of CD44 was considered as CD44-high.p<0.05 was considered significant. N0: without lymph node metastasis; N1: with lymph node metastasis; TNM: tumor, node, metastasis.

### Inducing ESCC Cells to Differentiation Reduces their Expression of CD44

Beside greater tumorigenicity, tumors generated from ESC1 cells exhibited less differentiation than those derived from ESC2 cells, as shown by light microscopy using H&E staining (Figure 1B). Meanwhile, the expression of p63, a marker of normal esophageal stem cells, was strongly expressed in ESC1 tumors (Figure 1B). The expression of CK13, a marker of differentiation esophageal epithelium, was down expressed in ESC1 tumors (Figure 1B). These results prompted us to analyze whether inducing the differentiation of ESCC cells can decrease the expression of CD44.

**Figure 4:**
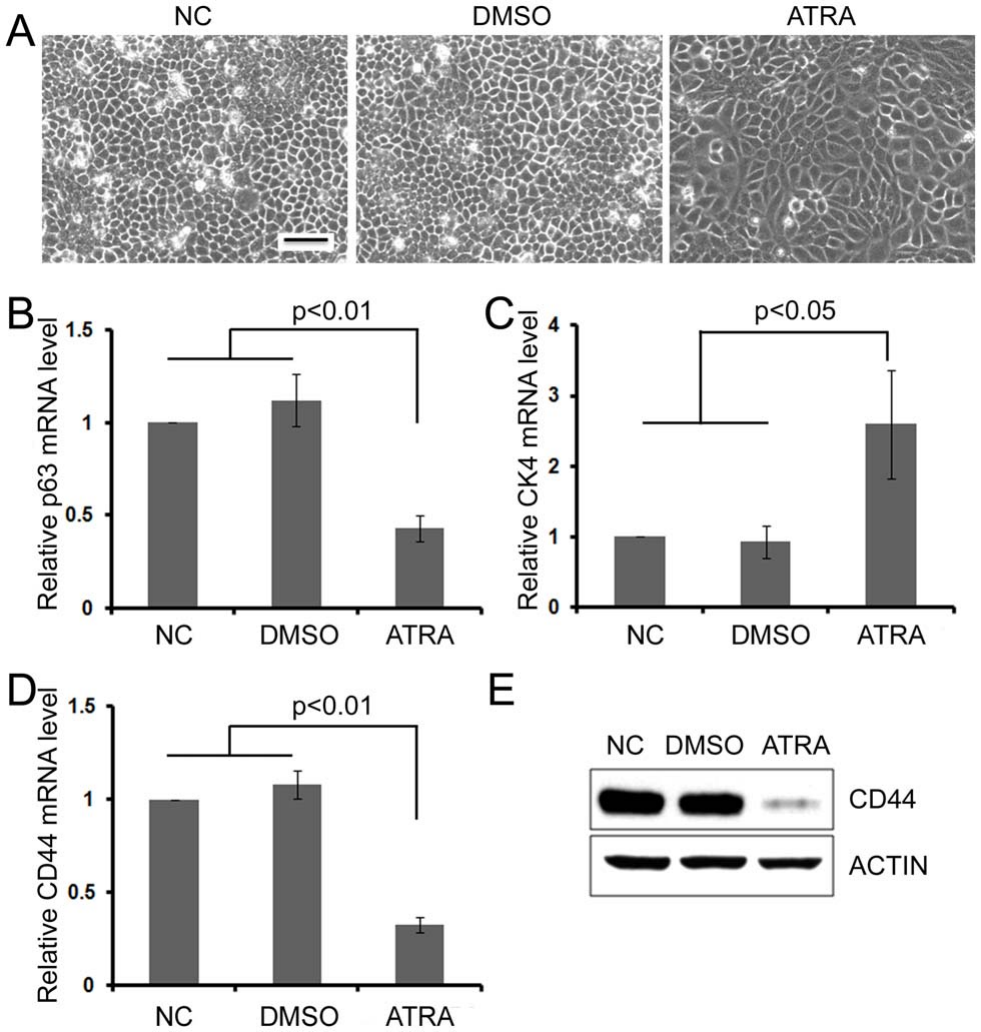
Differentiation of ESC1 downregulated expression of CD44. (A) Morphology of ESC1 cells treated with 20 µM ATRA for 5 days. (B–C) Real-time RT PCR of ATRA treated ESC1: p63 mRNA, an esophageal stemness marker (B); CK4, a differentiation marker (C). (D–E) Real-time RT PCR (D) and western blot (E) analysis of CD44 expression in ATRA-induced, differentiated ESC1 cells (20 µM, 5 days). Real-time RT PCR experiments were performed in triplicate and results were shown as mean ± SD. NC: blank control; DMSO: DMSO deluent treated cells; ATRA: ATRA treated cells; Scale bar: 50 micrometer.

After ESC1 or CaES17 cells were treated with 20 µM all-trans retinoic acid (ATRA), a widely used differentiation inducer, significant morphological alterations were observed. These ESC1 or CaES17 cells became larger and flatter after ATRA treatment (Figure 4A and Figure S1A). To verify the efficiency of ATRA-induced differentiation, we examined the mRNA expression of stemness and differentiation markers of esophageal epithelium cells by real-time PCR. Compared to controls, ATRA treatment significantly reduced the expression of p63 (stemness marker), while increasing the expression of CK4 (differentiation marker) (Figure 4B, C and Figure S1B, C). Also, the expression of CD44 at both the mRNA and protein level was significantly downregulated in these ATRA-induced differentiated ESCC cells (Figure 4D, E and Figure S1D, E).

### CD44H Enrich TICs in ESCC Cell Lines

**Figure 5:**
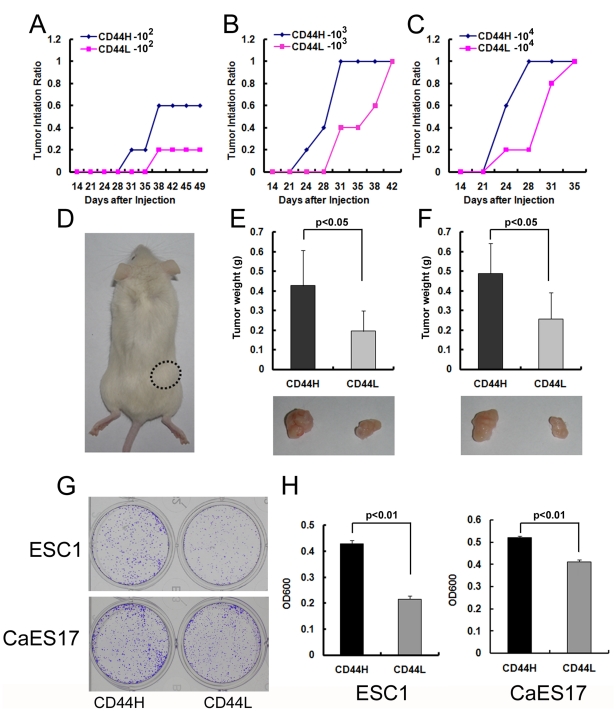
CD44H subpopulation was more tumorigenic than CD44L cells. (A–C) Tumorigenicity of 1×10^2^ (A), 1×10^3^ (B) or 1×10^4^ (C) sorted CD44H and CD44L ESC1 cells in NOD/SCID mice. (D) Representative NOD/SCID mice with subcutaneous tumors from either CD44H (circle dash line) or CD44L (no tumor formed) ESC1 cells at the dosage of 1×10^2^. (E–F) Average tumor weight of CD44H tumors and CD44L tumors at the dosage of either 1×10^3^ (E upper panel) or 1×10^4^ (F upper panel). Representative subcutaneous tumors derived from CD44H or CD44L ESC1 cells at the dosage of either 1×10^3^ (E lower panel) or 1×10^4^ (F lower panel). Data are generated from 5 mice in each group. (G) Representative photographs of the plates containing colonies derived from either 2000 CD44H or CD44L ESC1 or CaES17 cells. (H) Quantification of (G) by Crystal Violet staining. Colony formation experiments were performed in triplicate (mean ± SD).

To determine whether higher expression of CD44 can enrich TICs in ESCC cells, CD44H and CD44L cells were sorted from ESC1 by FACS and injected subcutaneously in NOD/SCID mice at different dosages. At 1×10^2^ cells, three out of 5 mice displayed visible tumors in the CD44H group at 5 weeks after injection. However, only one out of 5 mice at this cell dose generated visible tumors in the CD44L group (Figure 5A, D). Although all mice injected with higher cell doses of CD44H and CD44L cells (1×10^3^ and 1×10^4^) finally formed visible tumors, those from CD44H cells as compared to CD44L displayed a shorter latency for tumor development, and their tumors were a significantly larger (Figure 5B, C and E, F). A similar result was observed with Ec109 cells; 1×10^2^ sorted CD44H cells produced tumors in four of 5 injected mice, compared to two of 5 tumors in CD44L cell group (Table S3).

As previously reported, TICs reproduce tumors with similar characteristics as the primary ones. All the tumors derived from CD44H and unsorted ESC1 cells were pathologically analyzed by H&E staining, and no noticeable differences were observed (Figure S2A, B). Also, the percent and level of expression of CD44 within the tumors derived from either CD44H cells or parental ESC1 cells were similar as determined by flow cytometry (Figure S2D). We further isolated CD44H and CD44L cells from these first generate xenografts derived from CD44H cells to analyze their ability to reproduce serially transplantable tumors. As well as in the first generate xenografts, CD44H cells also shown stronger tumorigenicity in second generate xenografts (Figure S3).

Other than tumorigenicity, sphere formation and side population (SP) cells are considered as two important characteristics of TICs. However, we could not detect sphere formation in any of ESCC cells used in this study, even when the cells were cultured in sphere culture medium (DMEM/F12+B27+EGF+bFGF+Insulin) (data not show). SP cells were detected in CaES17 cells, but not in ESC1, ESC2 and Ec109 cells (data not show). So, no correlation was noted between degree of SP and tumorigenicity of the cell population.

**Figure 6:**
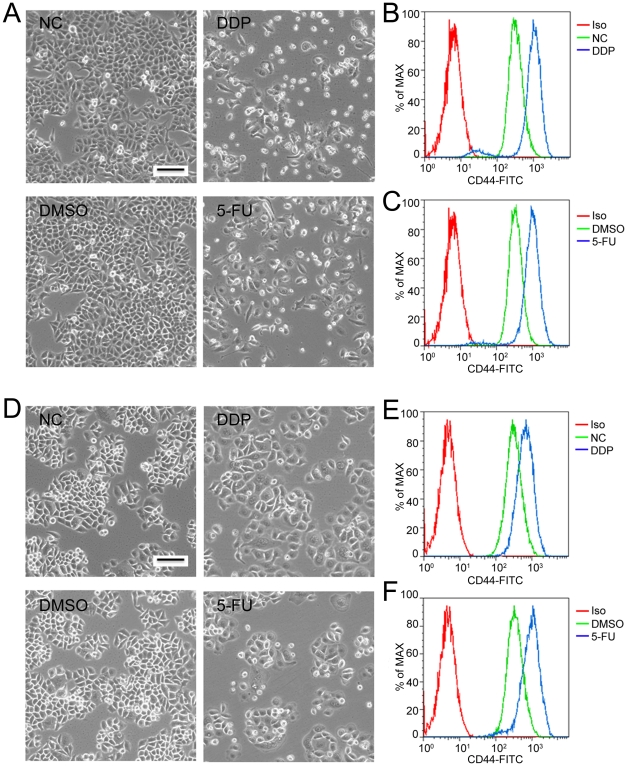
Chemotherapeutic drugs induced cell death in ESC1 and Ec109 cells and enriched for a CD44H population of cells. (A) Morphology of ESC1 cells treated with either 12.5 µg/ml DDP (cis-Diammineplatinum(II) dichloride, right upper panel) or 1.25 µg/ml 5-FU (5-Fluorouracil, right lower panel) for 2 days. (B–C) Representative flow cytometry analyses of CD44 expression in ESC1 cells after either DDP (B) or 5-FU (C) treatment. (D) Morphology of Ec109 cells treated with either 50 µg/ml DDP (right upper panel) or 2 µg/ml 5-FU (right lower panel) for 2 days. (E–F) Representative flow cytometry analyses of CD44 expression in Ec109 cells after either DDP (E) or 5-FU (F) treatment. Iso: isotype control; NC: blank control; DMSO: DMSO diluents treated cells; DDP: DDP treated cells; 5-FU: 5-FU treated cells; Scale bar: 50 micrometer.

Another characteristic of TICs is resistance to chemo- and radio-therapy. We treated ESC1 and Ec109 cells with traditional chemotherapeutic drugs, DDP (cis-Diammineplatinum(II) dichloride) and 5-Fu (5-Fluorouracil), and then analyzed the expression of CD44 by flow cytometry. DDP and 5-Fu treatment dramatically reduced the cell proportions at G2/M phase and induced cell death of ESC1 and Ec109 cells (Figure 6A, D and Table S4). In contrast, these same treatments markedly increased the percent of CD44H cells in both cell lines (Figure 6B, C and E, F), indicating that the CD44H population had a greater drug resistance to “cell- kill” by these two commonly used chemo-therapeutic drugs. To explore the mechanism, we analyzed the expression of some drug resistance related gene in both CD44H and CD44L cells. Two well known genes, ABCG2 and ABCA5 that involved in drug resistance in many types of tumor were overexpressed in CD44H cells (Figure S4).

The clonogenic capacity of CD44H and CD44L cells from ESC1 and CaES17 was also analyzed. Consistent with the previous reports concerning TICs in other tumors [13],[14], CD44H cells formed more and larger colonies than CD44L cells (Figure 5G, H). These may partially due to the overexpression of CDC2 in CD44H cells (Figure S4).

## Discussion

Although TICs have been widely reported in other tumors, few studies have focused on ESCC. SP cells have been found in several ESCC cell lines by staining with Hochest 33342, and these cells demonstrated greater tumorigenicity than non-SP cells [15]. p75NTR (CD271), a stem cell marker for normal human esophagus, was examined in ESCC cell lines. Previous study showed that p75NTR expressing ESCC cells were resistant to DDP and expressed higher levels of stem cell-associated genes [16]. These results suggested the existence of TICs in ESCC, but their identification and characterization remained elusive.

Using a similar strategy employed for identification of TICs markers in other tumors [17], we detected a group of candidate markers in two primary ESCC cells isolated from clinical specimens, and only CD44 expression correlated with tumorigenicity of these cells. Analysis of CD44 expression in an established immortalized esophageal epithelial cell line and ESCC cell lines further verified this correlation. CD44 is a widely used marker for TICs studies, especially in gastrointestinal tumors such as gastric and colon cancer [8],[19],[18],[20]. In these previous studies, CD44 positive TICs were enriched and characterized. However, cancer cells which highly expressed CD44 were also reported to be enriched for TICs in several other types of human cancers. For example, a subpopulation of breast cancer cells which highly expressed CD44, were characterized as TICs [21],[22]. Although we found the expression of CD44 correlated with tumorigenicity of ESCC cells, our flow cytometry results showed that nearly all the cells expressed varying intensities of CD44, including the immortalized esophageal epithelial cell line Het-1A. Therefore, we defined CD44H and CD44L samples based on the expression of CD44 in Het-1A (immortalized esophageal epithelial cell) and a low tumorigenic ESCC cell line (ESC2).

The heterogeneous expression of CD44 was previously reported in normal esophageal tissues [23],[24]. The strongest expression of CD44 occurred in the basal layer where normal esophageal stem cells are localized. Consistent with these results, we also found CD44 was positive in normal esophageal epithelial cells, and the strongest staining occurred in the basal layer. Overexpression of CD44 in ESCC has been noted previous, but a correlation between level of expression and prognosis was unclear [24],[25],[26],[27]. Although high expression of CD44v6, a variant of CD44, has been reported to correlate with invasive and metastatic ability of ESCC, we did not find a significant association between CD44 expression and clinicopathological indices in this study [26],[27]. However, we detected heterogeneous expression of CD44 in individual ESCC specimen. These results indicate that in most ESCC samples, cancer cells can be classified into different subpopulations based upon CD44 intensity of expression. Here, high levels of CD44 may be a feature not only of normal esophageal stem cells but also of TICs in ESCC.

The most important measure of TICs is their ability to initiate tumors in immunodeficient mice [28]. Therefore, we isolated CD44H and CD44L subpopulations from ESCC cells and injected them into NOD/SICD mice at serial dosages. A significant increase of tumorigenicity paralleled elevated expression of CD44 (CD44H), especially when low cell inoculums (100 cells) were injected. Consistent with these results, CD44H cells also produced more and larger colonies than CD44L cells in colony formation assays. Furthermore, the CD44H cells were more resistant to cell kill by traditional chemotherapeutic drugs, as shown by DDP and 5-FU significantly enriching the CD44H population of both ESC1 and Ec109 cells. Although sphere culture and SP cell analysis have been used to isolate or identify TICs in ESCC cell lines, neither method enriched TICs from cell lines examined in the present study. Sphere and SP cells have been detected in a limited number of cell lines. Also in some reports, SP cells did not show characteristics of TICs when compared with non-SP cells [29],[30]. Taken together, our results demonstrate that the CD44H subpopulation of cells represents the enriched TICs in ESCC cells.

A major purpose of identifying and characterizing TICs is to develop more effective strategies to eliminate cancers by targeting TICs. An attractive procedure is inducing differentiation of TICs; this approach can be called differentiation therapy. The well known use of ATRA in acute promyelocytic leukemia is a vivid illustration of this approach [31],[32],[33]. ATRA also is a widely used differentiation-inducer of stem cells [34],[35]. Previous data have shown that ATRA can induce differentiation of normal esophageal stem cells [36]. At the same time, ATRA can inhibit ESCC cell proliferation [37],[38],[39]. We found that ATRA can induce differentiation of ESC1 cells and dramatically downregulate expression of both mRNA and protein levels of CD44. These results suggest that ATRA can induce differentiation of TICs of ESCC suggesting a potential application of ATRA differentiation therapy for ESCC.

Although we show that isolating CD44H expressing cells can enrich for TICs in ESCC cells, function of this protein in TICs of ESCC is still unknown and deserves further study. Additionally, the fact that isolation of CD44H cells can enrich but cannot exclusively purify TICs in ESCC, suggests addition studies are needed to identify companion markers of TICs in ESCC.

## Materials and Methods

### Cell lines and Reagents

Two primary ESCC cell lines, ESC1 and ESC2, were recently derived from two ESCC patients treated at Zhongshan Hospital of Fudan University (Shanghai, China), after their written informed consent. Briefly, fresh cancer tissues were minced to 1 mm^3^ pieces and incubated in D-Hanks containing collagenase type I (1 mg/ml; Worthington Biomedical Corporation ) at 37°C for 10 to 12 hours. The digested ESCC tissue was incubated in ACK lysis buffer (TIANGEN) to remove red blood cells. After washing three times with D-Hanks, single cells were cultured in RPMI1640 supplemented with 10% fetal bovine serum, 10 units/ml penicillin/streptomycin, at 37°C in a humidified atmosphere containing 5% CO_2_. To enrich for the cancer cells, the adherent cells were digested with 0.05% trypsin containing 0.53 mM EDTA at 37°C for 30 seconds, resulting in the differential detachment of the fibroblasts with most cancer cells still remaining adherent.

HET-1A, an immortalized human esophageal epithelial cell line (ATCC) was cultured in Airway epithelial cell culture medium with supplements (PromoCell) with 10 units/ml penicillin/streptomycin. The other two ESCC cell lines, CaES17 and Ec109, were cultured similarly as ESC1 and ESC2.

Fluorescein isothiocyanate (FITC) conjugated CD44 and CD90 antibodies were purchased from BD Biosciences. FITC conjugated CD326 (EpCAM) and CD271 (p75NTR), and allophycocyanin (APC) conjugated CD133 antibodies were from Miltenyi Biotec. All comparable isotype control antibodies were from eBioscience. CD44 antibody used for Western Blot was purchased from Cell Signaling Technology. Actin antibody was from Santa Cruz Biotechnology. All-Trans Retinoic Acid (ATRA), cis-Diammineplatinum(II) dichloride (DPP) and 5-Fluorouracil (5-FU) were from Sigma-Aldrich.

### Flow cytometry and Fluorescence Activated Cell Sorting (FACS)

Cultured or treated cells were dissociated from culture plates by using trypsin-EDTA and centrifuged. Single cells were resuspended with D-Hanks containing 2% FBS, fluorochrome-conjugated antibody or compared isotype control antibody were added and the cells were incubated on ice for 30 minutes. After washing three times with D-Hanks containing 2% FBS, cells were resuspended with D-Hanks containing 2% FBS and 1 ug/ml propidium iodide (PI). Samples were analyzed or sorted by BD FACS Aria (BD Biosciences).

### Immunohistochemistry (IHC)

Paraffin embedded ESCC and normal mucosa specimens were obtained from 171 patients who were treated in the Department of Cardiothoracic Surgery of the First Affiliated Hospital of Shantou University Medical College and who provided written informed consent (Table S1). Immunohistochemistry was done as described previously [40]. The intensity of positive staining for CD44 was evaluated as negative, weak staining, moderate staining and strong staining. For statistical analysis, negative, weak and moderate staining for CD44 were considered as CD44-negative or -low expression, and strong staining of CD44 was considered as CD44-high expression.

### In vivo tumorigenicity assay

Six-week-old male nude mice or nonobese diabetic-severe combined immune deficient (NOD/SCID) mice were used in this study. All animal experiments were done in agreement with SIBS Guide for the Care and Use of Laboratory Animals and approved by Animal Care and Use Committee, Shanghai Institutes for Biological Sciences. For detecting tumor initiation ability, FACS sorted subsets of cells from ESC1 or Ec109 were mixed with matrigel (2∶1, volume/volume) and s.c. injected on both flanks of NOD/SCID mice. Tumor growth was monitored every three or four days. For other assays, cancer cells were injected into nude mice as described.

### Colony formation assay

2000 cells from both subsets of ESC1 after FACS were seeded in 6-well plates. After 10 days culture, cells were stained with Crystal Violet (0.5% Crystal Violet dissolved in 20% methanol). For cell number quantification, the stained cells were solubilized in 1% SDS and the absorbance at 600 nm was taken by a microplate reader.

### RNA isolation and Real-time PCR

Total RNA was extracted from cells using Trizol, according to the manufacturer's instructions. 2 µg RNA from each sample was processed directly to cDNA using the reverse transcription kit (Promega, Madison, WI). Amplification was done in a 15 µL reaction system with 0.2 µL SYBR Green. All of the reactions were performed in triplicate in an iCycler iQ System (Bio-Rad).

Primers used in this study were designed by software PRIMER5. The sequence of each primer is listed in Table S2.

### Western blot

Proteins were extracted from cells using RIPA buffer, and protein concentrations were quantified using Bradford reagent (Sigma) according to the manufacturer's instructions. Samples with equal amount of proteins were separated on 10% SDS-PAGE, and then transferred to Immobilon transfer membrane (Millipore, Bedford, MA) and immunoblotted with specific antibodies. All immunoblots were visualized by enhanced chemiluminescene.

### Statistical analysis

Statistical analysis was performed using SPSS 13.0 for windows. Statistically significant differences were determined by Student's test, one-way ANOVA and chi-square test, where appropriate, and defined as P<0.05.

## Supporting Information

Figure S1 **Differentiation of CaES17 downregulated expression of CD44.** (A) Morphology of CaES17 cells treated with 20 µM ATRA for 5 days. (B–C) Real-time RT PCR of ATRA treated CaES17: p63 mRNA, an esophageal stemness marker (B); CK4, a differentiation marker (C). (D–E) Real-time RT PCR (D) and western blot (E) analysis of CD44 expression in ATRA-induced, differentiated CaES17 cells (20 µM, 5 days). Real-time RT PCR experiments were performed in triplicate and results were shown as mean ± SD. NC: blank control; DMSO: DMSO deluent treated cells; ATRA: ATRA treated cells; Scale bar: 50 micrometer.
(TIF)

Figure S2 **CD44H cells formed similar tumors as parent cells.** (A–C) Representative H&E staining analyses of tumors derived from ESC1 CD44H and CD44L subpopulation cells and unsorted ESC1 cells. (D) Representative flow cytometry analyses of CD44 expression in ESC1 cells and tumors derived from ESC1 CD44H and CD44L subpopulation cells. ESC1: tumors derived from ESC1 cells; H-P1: tumors derived from ESC1 CD44H subpopulation cells; L-P1: tumors derived from ESC1 CD44L subpopulation cells; ESC1 Iso (red line): ESC1 tumor cells stained with isotype control antibody; ESC1 CD44 (green line): ESC1 tumor cells stained with CD44 antibody; H-P1 Iso (blue line): H-P1 tumor cells stained with isotype control antibody; H-P1 CD44 (brown line): H-P1 tumor cells stained with CD44 antibody; L-P1 Iso (pink line): L-P1 tumor cells stained with isotype control antibody; L-P1 CD44 (light blue line): L-P1 tumor cells stained with CD44 antibody;Scale bar: 50 micrometer.
(TIF)

Figure S3 **CD44H cells can reproduce serially transplantable tumors.** (A) Tumorigenicity of sorted CD44H and CD44L ESC1 cells in nude mice. (B) Tumorigenicity of sorted CD44H and CD44L ESC1 cells from the first generation tumors in nude mice. 1×10^4^ cells had been injected in both assays.
(TIF)

Figure S4 **Genes involved in drug resistance and cell growth were upregulsted in CD44H cells.** ABCG2, ABCB5 and CDC2 expression in CD44H and CD44L ESC1 cells were analyzed by Real-time RT PCR. Experiments were performed in triplicate and results were shown as mean ± SD.
(TIF)

Table S1 **Clinicopathologic features of patients with ESCC.**
(DOC)

Table S2 **Primers used for Real-Time PCR.**
(DOC)

Table S3 **Tumor initiation ability of CD44H and CD44L cells isolated from Ec109 cells.**
(DOC)

Table S4 **Cell death and cell cycle arrest of ESC1 and Ec109 after drug treatment.**
(DOC)
