# Supplementary figures and images for: Tumor Initiating Cells in Esophageal Squamous Cell Carcinomas Express High Levels of CD44
Source: PLoS One. 2011 Jun 24;6(6):e21419. doi: 10.1371/journal.pone.0021419 (PMC3123317; doi:10.1371/journal.pone.0021419)

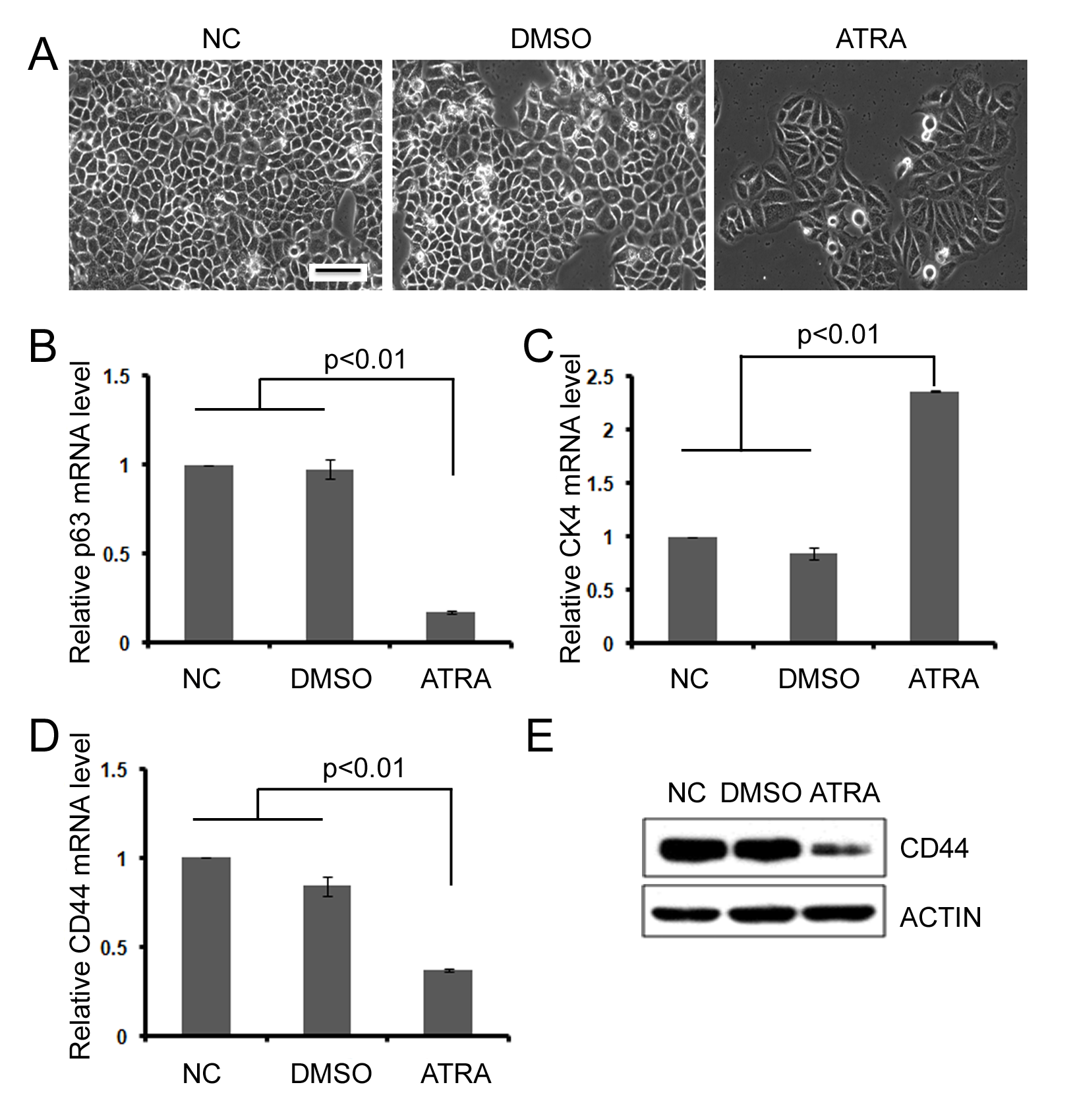

Supplement: Figure S1 — Differentiation of CaES17 downregulated expression of CD44. (A) Morphology of CaES17 cells treated with 20 µM ATRA for 5 days. (B–C) Real-time RT PCR of ATRA treated CaES17: p63 mRNA, an esophageal stemness marker (B); CK4, a differentiation marker (C). (D–E) Real-time RT PCR (D) and western blot (E) analysis of CD44 expression in ATRA-induced, differentiated CaES17 cells (20 µM, 5 days). Real-time RT PCR experiments were performed in triplicate and results were shown as mean ± SD. NC: blank control; DMSO: DMSO deluent treated cells; ATRA: ATRA treated cells; Scale bar: 50 micrometer. (TIF) [file pone.0021419.s001.tif]

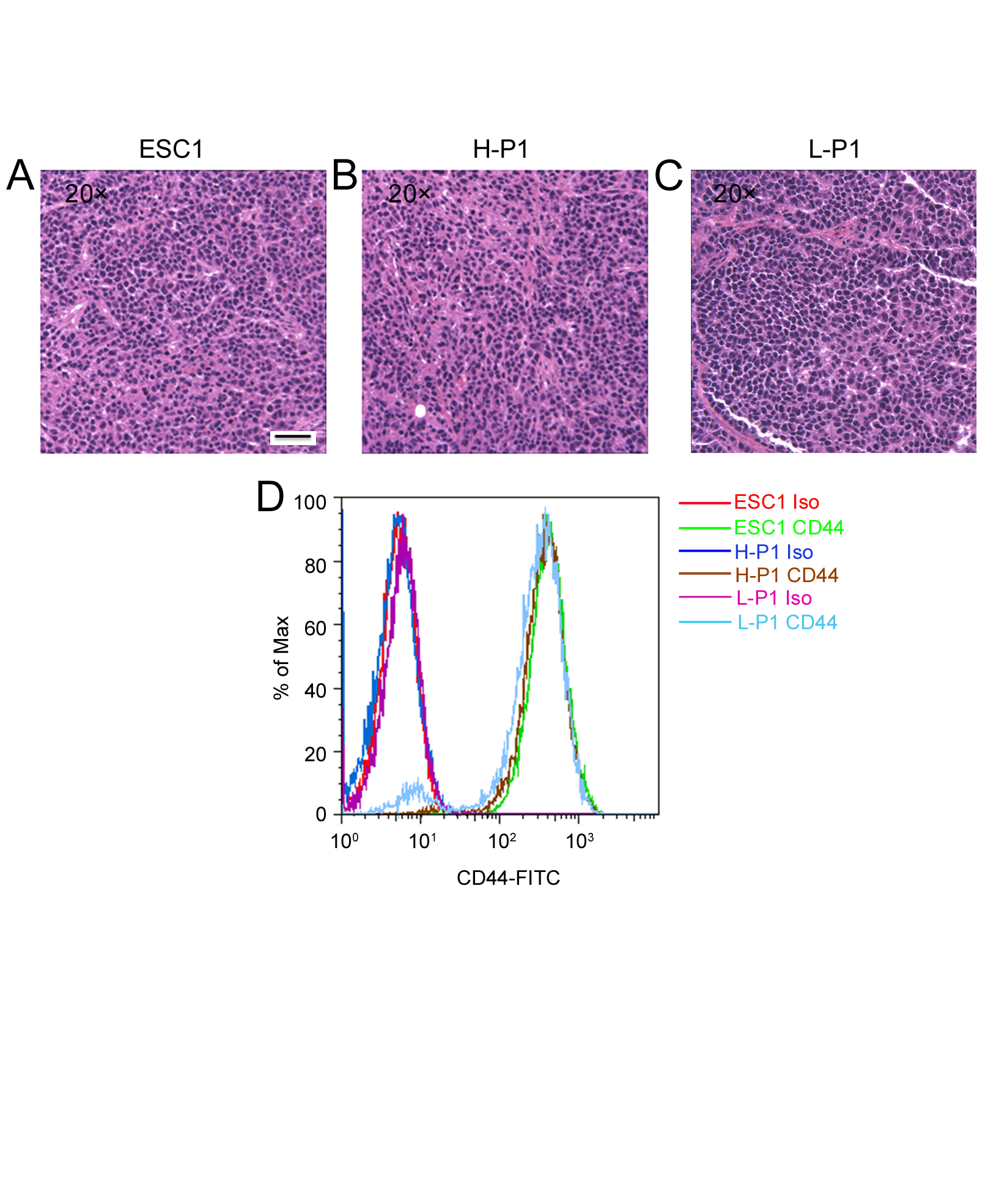

Supplement: Figure S2 — CD44H cells formed similar tumors as parent cells. (A–C) Representative H&E staining analyses of tumors derived from ESC1 CD44H and CD44L subpopulation cells and unsorted ESC1 cells. (D) Representative flow cytometry analyses of CD44 expression in ESC1 cells and tumors derived from ESC1 CD44H and CD44L subpopulation cells. ESC1: tumors derived from ESC1 cells; H-P1: tumors derived from ESC1 CD44H subpopulation cells; L-P1: tumors derived from ESC1 CD44L subpopulation cells; ESC1 Iso (red line): ESC1 tumor cells stained with isotype control antibody; ESC1 CD44 (green line): ESC1 tumor cells stained with CD44 antibody; H-P1 Iso (blue line): H-P1 tumor cells stained with isotype control antibody; H-P1 CD44 (brown line): H-P1 tumor cells stained with CD44 antibody; L-P1 Iso (pink line): L-P1 tumor cells stained with isotype control antibody; L-P1 CD44 (light blue line): L-P1 tumor cells stained with CD44 antibody;Scale bar: 50 micrometer. (TIF) [file pone.0021419.s002.tif]

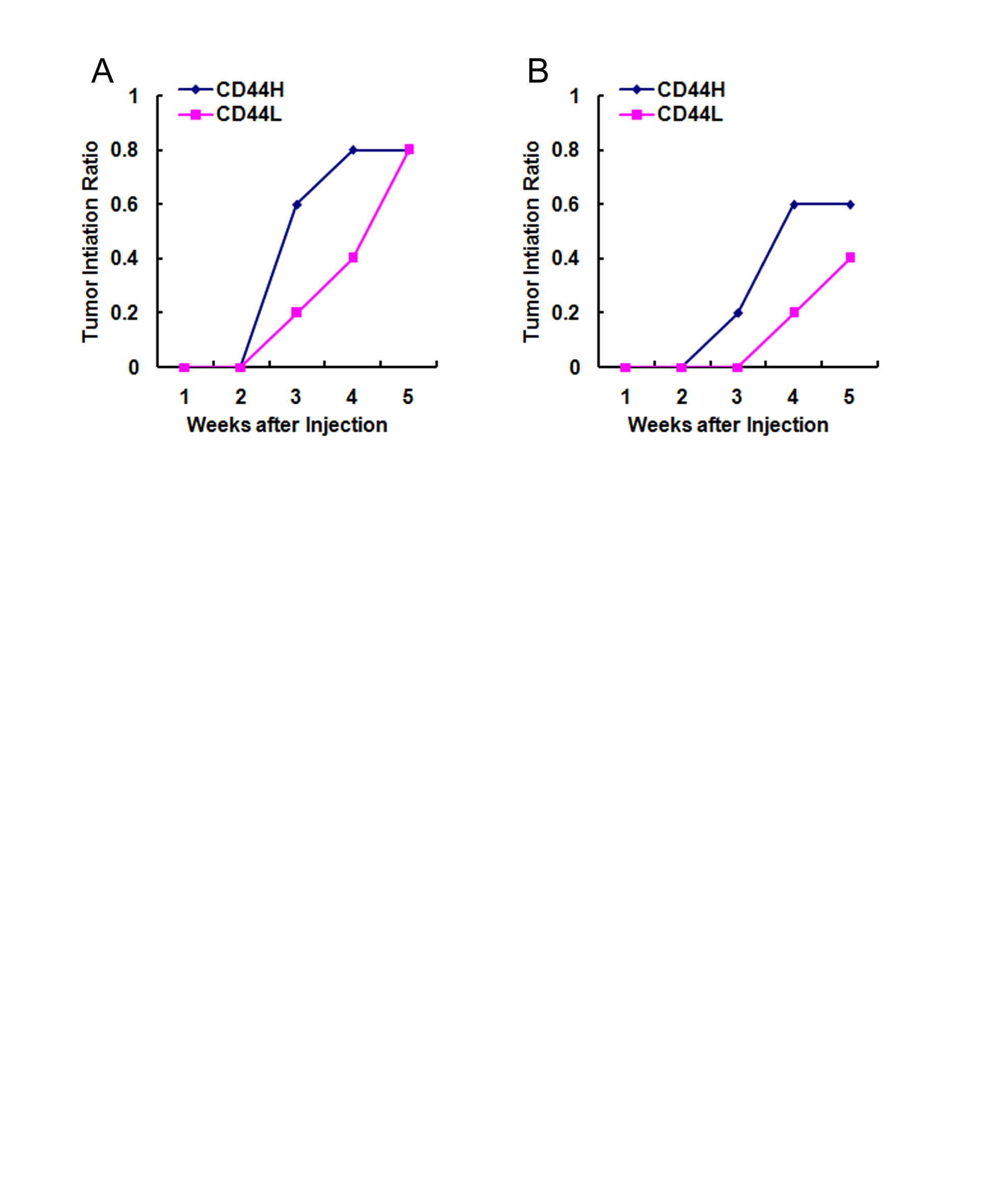

Supplement: Figure S3 — CD44H cells can reproduce serially transplantable tumors. (A) Tumorigenicity of sorted CD44H and CD44L ESC1 cells in nude mice. (B) Tumorigenicity of sorted CD44H and CD44L ESC1 cells from the first generation tumors in nude mice. 1×10^4^ cells had been injected in both assays. (TIF) [file pone.0021419.s003.tif]

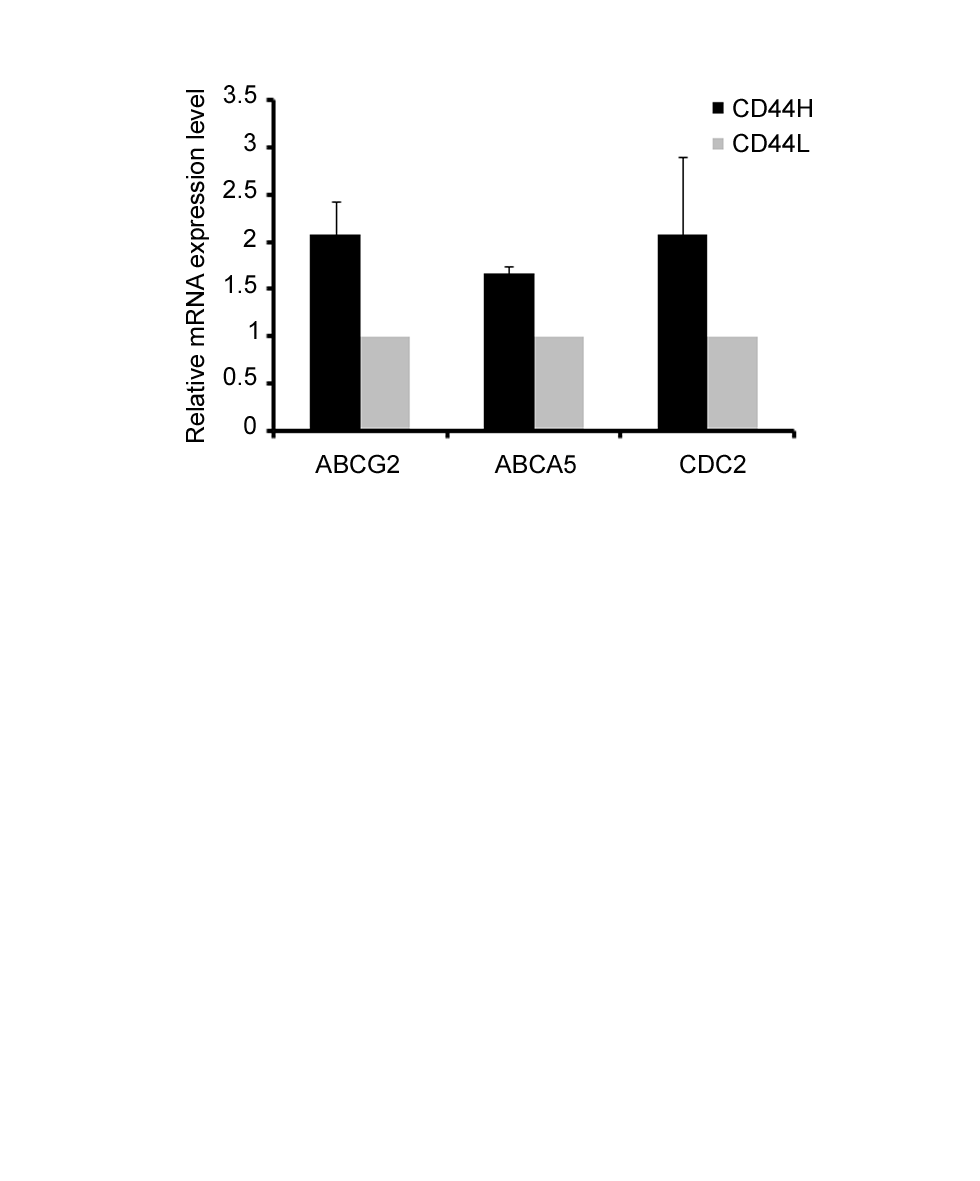

Supplement: Figure S4 — Genes involved in drug resistance and cell growth were upregulsted in CD44H cells. ABCG2, ABCB5 and CDC2 expression in CD44H and CD44L ESC1 cells were analyzed by Real-time RT PCR. Experiments were performed in triplicate and results were shown as mean ± SD. (TIF) [file pone.0021419.s004.tif]
