# Supplementary material for: Tumor Initiating Cells in Esophageal Squamous Cell Carcinomas Express High Levels of CD44
Source: PLoS One. 2011 Jun 24;6(6):e21419. doi: 10.1371/journal.pone.0021419 (PMC3123317; doi:10.1371/journal.pone.0021419)
Supplement: Table S1 — Clinicopathologic features of patients with ESCC. (DOC) [file pone.0021419.s005.doc]

**Table S1.** Clinicopathologic features of patients with ESCC

| **Parameters** | **IHC (***n***= 117)** |
| --- | --- |
| Mean age in years (range) | 57.8 (35–75) |
| ＜55 | 63 (37%) |
| ≥55 | 108 (63%) |
| Gender |  |
| Male | 128 (75%) |
| Female | 43 (25%) |
| Regional lymph nodes |  |
| N0 | 91 (53%) |
| N1 | 80 (47%) |
| TNM staging |  |
| I/IIa | 91 (53%) |
| IIb/III/IV | 80 (47%) |
| Grade of differentiation |  |
| Well | 48 (28%) |
| Moderate | 103 (60%) |
| Poor | 20 (12%) |

N0: without lymph node metastasis; N1: with lymph node metastasis; TNM: tumor, node, metastasis.
