# Supplementary material for: Tumor Initiating Cells in Esophageal Squamous Cell Carcinomas Express High Levels of CD44
Source: PLoS One. 2011 Jun 24;6(6):e21419. doi: 10.1371/journal.pone.0021419 (PMC3123317; doi:10.1371/journal.pone.0021419)
Supplement: Table S2 — Primers used for Real-Time PCR. (DOC) [file pone.0021419.s006.doc]

**Table S2. Primers used for Real-Time PCR**

| **Gene** | **Primer sequence** |
| --- | --- |
| p63 | GAAAGCAGCAAGTTTCGGAC  TTTCATAAGTCTCACGGCCC |
| CK4 | ACCACCACCCTGAACAAGAG  GATGAGCCCCAGAGACAGAG |
| CD44 | GAGCATCGGATTTGAGA  CATACTGGGAGGTGTTGG |
| POLR2A | GCAGGACGTAATAGAGG  CGGACACGACCATAGA |

[POLR2A](http://www.ncbi.nlm.nih.gov/gene/5430)**:** polymerase (RNA) II (DNA directed) polypeptide A (used as internal control)**.**
