# Supplementary material for: Tumor Initiating Cells in Esophageal Squamous Cell Carcinomas Express High Levels of CD44
Source: PLoS One. 2011 Jun 24;6(6):e21419. doi: 10.1371/journal.pone.0021419 (PMC3123317; doi:10.1371/journal.pone.0021419)
Supplement: Table S3 — Tumor initiation ability of CD44H and CD44L cells isolated from Ec109 cells. (DOC) [file pone.0021419.s007.doc]

**Table S3. Tumor initiation ability of CD44H and CD44L cells isolated from Ec109 cells**

| **Cell dosage** | **Tumor initiation ability** | |
| --- | --- | --- |
|  | **CD44H** | **CD44L** |
| 10^2^ | 4/5 | 2/5 |
| 10^3^ | 5/5 | 5/5 |
| 10^4^ | 5/5 | 5/5 |

FACS sorted CD44H and CD44L cells from Ec109 were mixed with matrigel (2:1, volume/volume) and s.c. injected on both flanks of NOD/SCID mice. Tumors were allowed to grow for seven weeks, and then visible tumors were counted.
