# Supplementary material for: Tumor Initiating Cells in Esophageal Squamous Cell Carcinomas Express High Levels of CD44
Source: PLoS One. 2011 Jun 24;6(6):e21419. doi: 10.1371/journal.pone.0021419 (PMC3123317; doi:10.1371/journal.pone.0021419)
Supplement: Table S4 — Cell death and cell cycle arrest of ESC1 and Ec109 after drug treatment. (DOC) [file pone.0021419.s008.doc]

**Table S4. Cell death and cell cycle arrest of ESC1 and Ec109 after drug treatment**

|  | **Cell proportion in different phases (%)** | | | |
| --- | --- | --- | --- | --- |
|  | **Sub-G0** | **G0/G1** | **S** | **G2/M** |
| **ESC1** |  |  |  |  |
| **NC** | 1.85 | 62.02 | 11.29 | 24.85 |
| **DMSO** | 1.88 | 64.42 | 11.06 | 22.64 |
| **DDP** | 28.25 | 29.99 | 58.70 | 13.06 |
| **5-Fu** | 5.20 | 25.61 | 52.34 | 16.86 |
| **Ec109** |  |  |  |  |
| **NC** | 2.05 | 51.63 | 13.28 | 33.04 |
| **DMSO** | 2.88 | 48.43 | 13.54 | 35.15 |
| **DDP** | 8.09 | 23.97 | 35.98 | 31.96 |
| **5-Fu** | 7.64 | 21.85 | 52.29 | 18.22 |

After 2day of different treatments, ESC1 and Ec109 cells were analyzed by PI staining assay. NC: blank control; DMSO: DMSO diluents treated cells; DDP: DDP treated cells; 5-FU: 5-FU treated cells.
